# Comprehensive analysis of BTNL9 as a prognostic biomarker correlated with immune infiltrations in thyroid cancer

**DOI:** 10.1186/s12920-023-01676-8

**Published:** 2023-10-05

**Authors:** Luyao Zhang, Shuang Yu, Shubin Hong, Xi Xiao, Zhihong Liao, Yanbing Li, Haipeng Xiao

**Affiliations:** Department of Endocrinology, The First Affiliated Hospital, Sun Yat-sen University, Guangzhou, 510080 China

**Keywords:** *BTNL9*, Prognosis, Immune infiltration, Thyroid cancer, Biomarker

## Abstract

**Background:**

Thyroid cancer (THCA) is the most common type of endocrine cancers, and the disease recurrences were usually associated with the risks of metastasis and fatality. Butyrophilin-like protein 9 (BTNL9) is a member of the immunoglobulin families. This study investigated the prognostic role of BTNL9 in THCA.

**Methods:**

Gene enhancers of *BTNL9* were identified by interrogating H3K27ac ChIP-seq and RNA-seq data of papillary thyroid cancer (PTC) and benign thyroid nodule (BTN) tissues. Meanwhile, *BTNL9* expression level was verified by qRT-PCR in 30 pairs of primary THCA and adjacent normal tissues. Clinicopathological and RNA sequencing data were obtained from The Cancer Genome Atlas (TCGA) and Genotype-Tissue Expression (GTEx) to analyze the relations between *BTNL9* expression and immune cell infiltration, chemokines/cytokines, immune checkpoint genes, clinical parameters and prognosis values. Besides, survival analysis combining *BTNL9* expression and immune cell infiltration scores was conducted. Functional enrichment analysis was performed to investigate the potential biological mechanisms. Cox regression analyses were used to explore independent clinical indicators, and a nomogram model incorporating *BTNL9* expression with clinical parameters was established.

**Results:**

*BTNL9* showed significantly stronger H3K27ac modifications in BTN than PTC tissues at the promoter region (chr5: 181,035,673–181,047,436) and gene body (chr5: 181,051,544–181,054,849). The expression levels of *BTNL9* were significantly down-regulated in THCA samples compared to normal tissues, and were strongly associated with different tumor stages, immune cell infiltrations, chemokines/cytokines and immune checkpoint genes in THCA. Functional enrichment analyses indicated that BTNL9 was involved in immune-related and cancer-related pathways. The Kaplan–Meier analysis showed lower *BTNL9* expression was associated with poorer progression-free interval (PFI). *BTNL9* expression and pathologic stages were independent prognostic indicators of PFI in THCA.

**Conclusions:**

The results implied an important role of BTNL9 in the tumor progression, with the possibility of serving as a novel prognostic biomarker and a potential therapeutic target for THCA.

**Supplementary Information:**

The online version contains supplementary material available at 10.1186/s12920-023-01676-8.

## Background

Thyroid cancer (THCA) is the most common type of endocrine cancers, taking up about 90% of all endocrine tumors and 2% of all systemic malignancies [1, 2], and it ranked the fifth most prevalent tumor in women [3]. Generally, THCA could be categorized into different subtypes based on histologic features, among which papillary thyroid cancer (PTC) accounts for over 80% of the cases [2]. About one third of THCA patients might evolve with disease recurrences, facing the risks of metastasis and THCA-related fatality [4, 5]. Moreover, with the development of imaging technology and increased use of neck ultrasound for the routine detection of thyroid neoplasms, the thyroid nodules were detected in approximately 50% adults [6]. Thus, it is of great significance to differentiate between malignant and benign nodules so as to reduce overdiagnosis and unnecessary treatment for people with thyroid nodules. However, the fine-needle aspiration guided by ultrasound could only help to make differentiation diagnosis in about 75% nodules [7, 8], and the remaining indeterminate nodules hindered the medical management of these patients. Therefore, it is of clinical significance to find new molecular biomarkers for the differentiation and prognosis evaluation.

Butyrophilin-like protein 9 (BTNL9) is a member of the immunoglobulin butyrophilin and butyrophilin-like (BTNL) families, modulating the T cell response and impacting inflammatory disorders and cancers [9]. It is reported that recombinant BTNL9–Fc could bind with activated T cells, B cells, dendritic cells and macrophages [10, 11]. In immune cells, BTNL9 was mostly expressed in B cells yet its functions were not well understood. Few studies reported the roles and mechanisms of BTNL9 in cancer. BTNL9 was reported to have lower expression levels in various cancers compared to normal tissues, including colon cancer, lung adenocarcinoma, osteosarcoma, breast cancer and uveal melanoma [12–16]. However, the prognosis value and immunology of BTNL9 in THCA have not been elucidated.

In this study, the H3K27ac modification and RNA expression differences of *BTNL9* gene between PTC and benign thyroid nodules (BTN) were characterized, revealing the epigenetic modifications on gene transcription spectrum. The expression level of *BTNL9* was further validated in 30 pairs of THCA and corresponding normal tissues. Furthermore, comprehensive analyses were performed including the correlation between *BTNL9* expression and clinical parameters, prognostic value, immune cell infiltration, immune biomarkers and immune checkpoints, with functional enrichment analyzed on differentially expressed genes (DEGs) to explore the functions and potential mechanisms of BTNL9, raising the possibility of BTNL9 serving as a novel prognostic indicator and an immunotherapeutic target in THCA.

## Methods

### Data collection and analysis

Gene mRNA expression data with clinical information was downloaded from the TCGA database (https://portal.gdc.cancer.gov/) and the GTEx projects (Genotype-Tissue Expression, https://gtexportal.org/). Statistical analysis was performed using R software (v. 3.6.3). The differential expression analysis of *BTNL9* gene was analyzed with GEPIA online database (Gene Expression Profiling Interactive Analysis) (http://gepia.cancer-pku.cn/), which is a comprehensive platform with TCGA and GTEx data analyzed for cancer research [17].

### Survival prognosis analysis

The Kaplan-Meier plotter (http://kmplot.com), an online interactive platform, which could estimate the effects of gene expression on prognosis in various cancers [18], was used to investigate the correlation between *BTNL9* expression and prognosis in THCA. Besides, subgroup survival analysis was performed using different clinical parameters. The hazard ratio (HR) and log-rank *p*-value were estimated.

Cox proportional hazards models were performed to evaluate prognostic indicators of THCA. Univariate and multivariate Cox regression analyses were performed with survival package (v. 3.2–10). Nomogram model incorporating *BTNL9* expression and clinical indicators were generated using rms (v. 6.2-0) and survival package.

### Immune infiltration analysis

The Tumor Immune Estimation Resource (TIMER2.0) (http://timer.cistrome.org/) [19], a comprehensive online platform for tumor-infiltrating assessment, was used to analyze the immune cell infiltrations in THCA. The associations between *BTNL9* gene and various chemokines/cytokines were evaluated via TIMER database.

The relationships between *BTNL9* expression and infiltration levels of 24 types of immune cells [20] were analyzed via ssGSEA algorithm using GSVA package (v. 1.34.0) [21]. The associations between *BTNL9* expression and immune checkpoint genes were visualized by heatmap plots using ggplot2 R package (v. 3.3.3). Kaplan-Meier survival analysis with the combined data of *BTNL9* expression and immune cell infiltration scores was conducted with survminer (v. 0.4.9) and survival (v. 3.2–10) packages.

### Gene enrichment analysis

The Linked Omics database (http://www.linkes.org/login.php) is a web-based tool for the analysis of multi-omics data across different cancer types [22]. The genes associated with *BTNL9* were analyzed and visualized by the Linked Omics.

The DEGs were analyzed by DESeq2 package (v.1.26.0) with the median *BTNL9* expression level set as cut-off value to define low and high expression subgroups. DEGs with |Log2(Fold Change)| >1 and adjusted *p*<0.05 were then subjected to functional enrichment analysis. The Gene Ontology (GO) terms annotation and Kyoto Encyclopedia of Genes and Genomes (KEGG) pathway analysis were performed with ClusterProfiler package (v. 3.14.3) [23]. Gene set enrichment analysis (GSEA) [24] using predefined gene sets from MSigDB Collections (https://www.gsea-msigdb.org/gsea/msigdb/collections.jsp) was conducted, and results with false discovery rate (FDR) < 0.25 and adjusted *p*<0.05 were defined as significant. The SRTING Database (https://cn.string-db.org/), an online tool for protein-protein interaction networks analysis, was used to investigate the integrated network of BTNL9.

### H3K27ac ChIP-seq and RNA-seq data

The detailed protocols of ChIP-seq and RNA-seq were reported in our previous article [25]. The data can be found at Genome Sequence Archive in National Genomics Data Center, China National Center for Bioinformation/Beijing Institute of Genomics, Chinese Academy of Sciences (Accession number HRA000779).

### Real-time quantitative PCR analysis

30 pairs of excised THCA and paracancerous thyroid tissues were obtained from the First Affiliated Hospital of Sun Yat-sen University after institutional review board approval and informed patient consent. Total mRNAs of tissues were extracted using RNAsimple Total RNA Kit (#DP419; TIANGEN Biotech). 500ng mRNA was used as template for reverse transcription with PrimeScript RT Master Mix (RR036A; Takara), and SYBR Green Master Mix (A25742; Thermo Fisher Scientific) was used to detect cDNA amplification. *GAPDH* was used to normalize gene expression. The primers for *BTNL9* were as follows: forward 5’- ATGGTGGACCTCTCAGTCTCC-3’, reverse 5’-GCCAGGATGGGATACTCAGG-3’. *GAPDH* primers: forward 5’- ACTTCAACAGCGACACCCACTC-3’, reverse 5’- TCTCTTCCTCTTGTGCTCTTGCT-3’. Relative expression of genes was calculated using the 2^− ΔΔCT^ method.

### Statistics

Statistical analysis was performed using R software (v 3.6.3). One-way ANOVA test and student *t* test were adopted in comparing the clinical features of high-expression and low-expression groups; Chi-Square test, Wilcoxon and Kruskal-Wallis tests were used when needed. Spearman’s correlation coefficient was used to evaluate the correlations with immune cell infiltrations. Statistical significance was defined as *p* < 0.05.

## Results

### Differentiated *BTNL9* expression between thyroid cancer and normal tissues

**Fig. 1:**
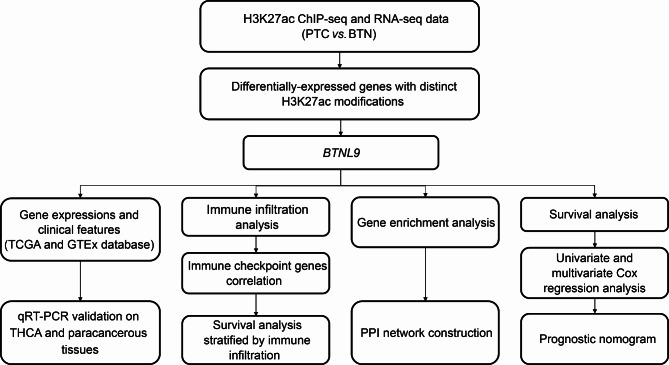
Flow chart shows the analysis procedure of the study. PTC, papillary thyroid carcinoma; BTN, benign thyroid nodule; TCGA, The Cancer Genome Atlas; GTEx, Genotype-Tissue Expression; THCA, thyroid carcinoma

**Fig. 2:**
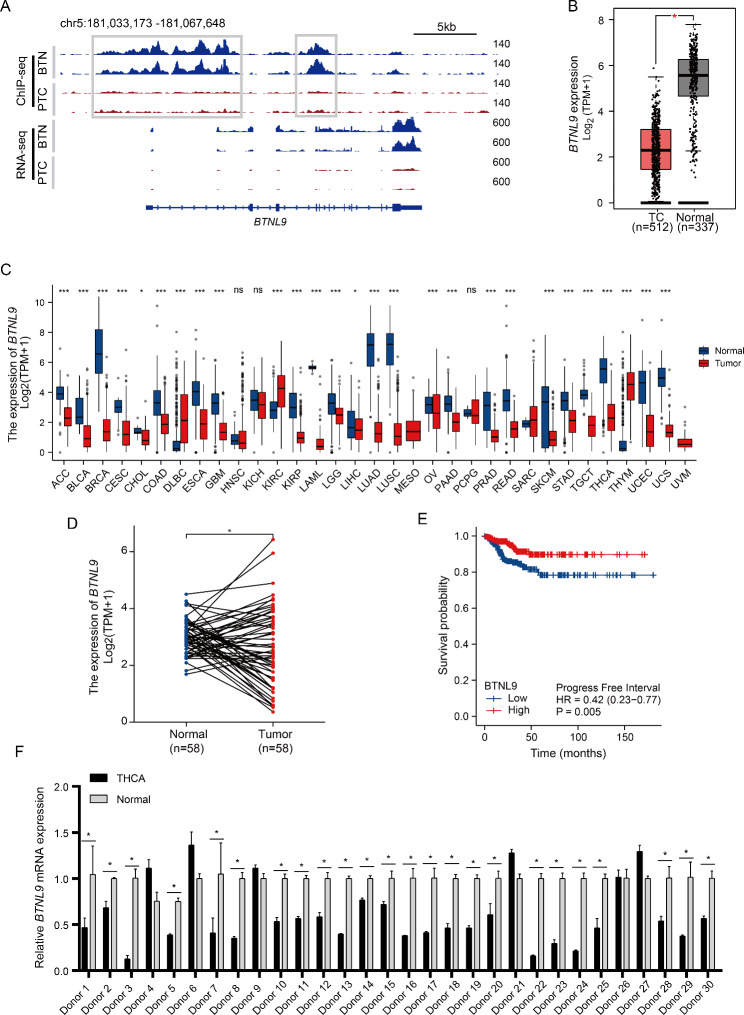
Expression levels of *BTNL9* gene in THCA. **(A)** Gene tracks of BTN-specific enhancers (chr5:181,035,673–181,047,436; chr5:181,051,544–181,054,849). The adjacent gene *BTNL9* showed much stronger signals in H3K27ac ChIP-seq and RNA-seq tracks in BTN tissues than PTC tissues. **(B)** The expression level of *BTNL9* was significantly lower in THCA compared with normal samples based on TCGA and GTEx datasets. **(C)***BTNL9* expression in different cancer types from TCGA and GTEx datasets. **(D)** Decreased expression of *BTNL9* in THCA compared with the matched normal tissues from TCGA datasets. **(E)** Higher level of *BTNL9* transcription is associated with more favorable PFI in patients with THCA. Analysis was performed with TCGA (THCA) datasets.** (F)** Relative *BTNL9* mRNA expression levels in 30 pairs of THCA and paired paracancerous tissues. Expression analysis was normalized against GAPDH. *p < 0.05, **p < 0.01, ***p < 0.001. THCA, thyroid carcinoma; PTC, papillary thyroid carcinoma; BTN, benign thyroid nodule; PFI, progress-free interval; TCGA, The Cancer Genome Atlas; GTEx, Genotype-Tissue Expression. ACC, adrenocortical carcinoma; BLCA, bladder urothelial carcinoma; BRCA, breast invasive carcinoma; CESC, cervical and endocervical cancers; CHOL, cholangiocarcinoma; COAD, colon adenocarcinoma; DLBC, lymphoid neoplasm diffuse large B-cell lymphoma; ESCA, esophageal carcinoma; GBM, glioblastoma multiforme; HNSC, head and neck squamous cell carcinoma; KICH, kidney chromophobe; KIRC, kidney renal clear cell carcinoma; KIRP, kidney renal papillary cell carcinoma; LAML, acute myeloid leukemia; LGG, brain lower grade glioma; LIHC, liver hepatocellular carcinoma; LUAD, lung adenocarcinoma; LUSC, lung squamous cell carcinoma; MESO, mesothelioma; OV, ovarian serous cystadenocarcinoma; PAAD, pancreatic adenocarcinoma; PCPG, pheochromocytoma and paraganglioma; PRAD, prostate adenocarcinoma; READ, rectum adenocarcinoma; SARC, sarcoma; SKCM, skin cutaneous melanoma; STAD, stomach adenocarcinoma; TGCT, testicular germ cell tumors; THYM, thymoma; UCEC, uterine corpus endometrial carcinoma; UCS, uterine carcinosarcoma; UVM, uveal melanoma

A flow chart illustrating the analysis procedure of the study is shown in Fig. 1. H3K27ac ChIP-seq and RNA-seq data of PTC and BTN were integrated to map the enhancers of *BTNL9* gene. As shown in Fig. 2A, BTN samples had stronger H3K27ac signals than PTC samples at the promoter (chr5: 181,035,673–181,047,436) and gene body (chr5: 181,051,544–181,054,849) of *BTNL9*. The RNA-seq data revealed that *BTNL9* expression was higher in BTN than PTC samples. Additionally, qRT-PCR on 30 pairs of primary samples verified that *BTNL9* expression levels were significantly down-regulated in THCA than matched paracancerous tissues frequently (Fig. 2F). Consistently, when analyzing the data from TCGA and GTEx, significantly lower expression levels of *BTNL9* in THCA than normal controls (Fig. 2B) and matched normal samples (n = 58) were observed (Fig. 2D). The differences in epigenetic H3K27ac modification and expression indicated that *BTNL9* might be a potential diagnostic biomarker for the differentiation between THCA and benign nodules.

*BTNL9* expression levels in other 32 types of cancer were analyzed with TCGA and GTEx datasets (Fig. 2C). It revealed that *BTNL9* expression was significantly down-regulated in various cancers, including adrenocortical carcinoma (ACC), bladder urothelial carcinoma (BLCA), breast invasive carcinoma (BRCA), cervical and endocervical cancer (CESC), cholangiocarcinoma (CHOL), colon adenocarcinoma (COAD), esophageal carcinoma (ESCA), glioblastoma multiforme (GBM), kidney renal clear cell carcinoma (KIRC), kidney renal papillary cell carcinoma (KIRP), acute myeloid leukemia (LAML), brain lower grade glioma (LGG), liver hepatocellular carcinoma (LIHC), lung adenocarcinoma (LUAD), lung squamous cell carcinoma (LUSC), ovarian serous cystadenocarcinoma (OV), pancreatic adenocarcinoma (PAAD), prostate adenocarcinoma (PRAD), rectum adenocarcinoma (READ), skin cutaneous melanoma (SKCM), stomach adenocarcinoma (STAD), testicular germ cell tumor (TGCT), uterine corpus endometrial carcinoma (UCEC), and uterine carcinosarcoma (UCS).

### Prognostic analysis and clinical relevance of *BTNL9* expression in thyroid cancer

**Fig. 3:**
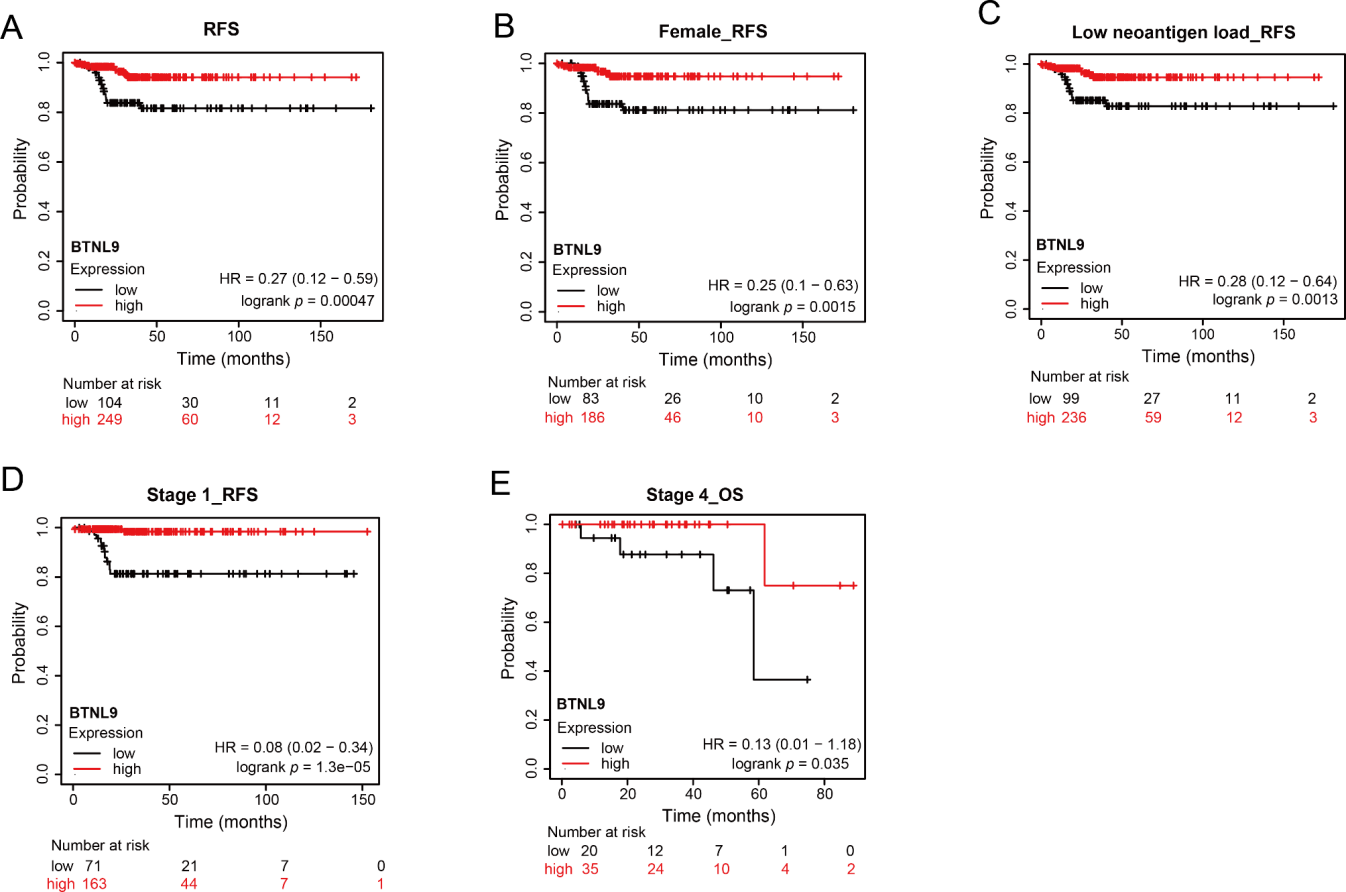
Kaplan-Meier survival curves for patients with THCA from KM plotter web-based tool. **(A)** Higher expression of *BTNL9* was associated with better RFS in patients with THCA. **(B)** Subgroup analysis indicated that *BTNL9* expression was associated with more favorable RFS in female THCA patients. **(C)***BTNL9* expression was associated with more favorable RFS in THCA patients with low neoantigen load. **(D)** Higher expression of *BTNL9* was associated with better RFS in patients with Stage 1 THCA. **(E)** Higher expression of *BTNL9* was associated with better OS in patients with Stage 4 THCA. RFS, relapse free survival; OS, overall survival; THCA, thyroid carcinoma

Kaplan–Meier survival curves demonstrated that high *BTNL9* expression was correlated with favorable progression free interval (PFI) (Fig. 2E) and relapse-free survival (RFS) (Fig. 3A) in patients with THCA based on TCGA datasets. Specifically, subgroup survival analysis showed that increased *BTNL9* expression was associated with favorable RFS in female patients (Fig. 3B), tumor with low neoantigen load (Fig. 3C) and patients with stage 1 THCA (Fig. 3D). In stage 4 THCA subgroup, greater *BTNL9* expression was related to better overall survival (OS) outcomes (Fig. 3E). These results suggested the potential of *BTNL9* serving as a prognostic biomarker in THCA.

**Fig. 4:**
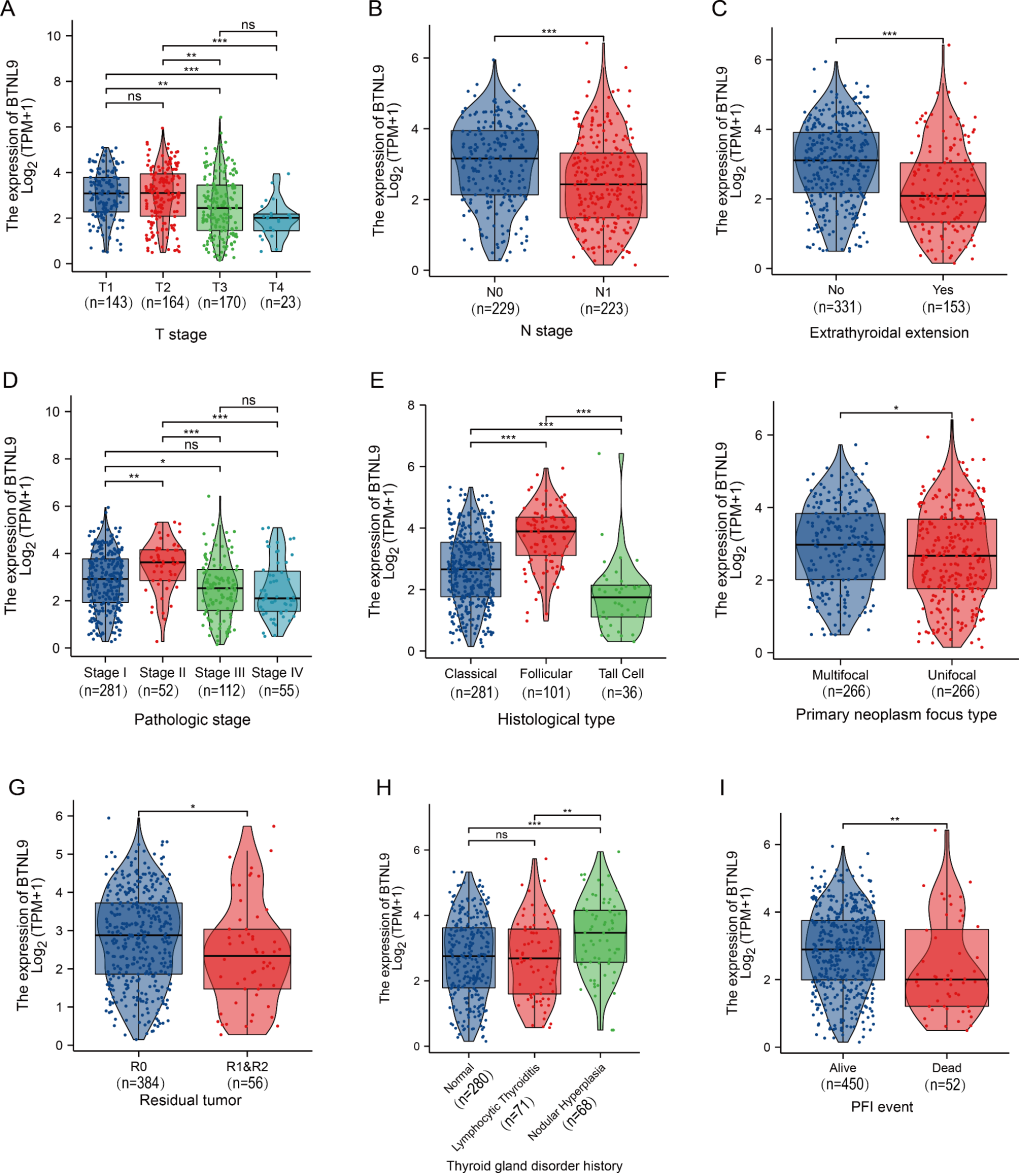
Association between *BTNL9* expression and clinical features in patients with THCA based on TCGA datasets. *BTNL9* expression levels were stratified by **(A)** tumor T stage, **(B)** tumor N stage, **(C)** extrathyroidal extension, **(D)** pathologic stage, **(E)** histological type, **(F)** primary neoplasm focus type, **(G)** residual tumor, **(H)** thyroid gland disorder history and **(I)** PFI event. TCGA, The Cancer Genome Atlas; THCA, thyroid carcinoma; PFI, progress-free interval. *p < 0.05, **p < 0.01, ***p < 0.001

To analyze the relationships between *BTNL9* expression and clinical characteristics in patients with THCA, 502 patients from TCGA datasets were categorized into high and low expression groups with the mean value of *BTNL9* expression set as dividing threshold (Table 1). It showed that *BTNL9* expression was significantly associated with different clinical T stages, N stages, extrathyroidal extension, pathologic stages, histological types, primary neoplasm focus types, residual tumors, thyroid gland disorder history and disease progression (Fig. 4) in THCA patients.

**Table 1:** Correlation between *BTNL9* expression and clinical characteristics in patients with thyroid cancer

Characteristics	*BTNL9* expression		*p* value
	Low (n = 251)	High (n = 251)	
Age, n (%)			1.000
≤ 45	118 (23.5%)	118 (23.5%)	
> 45	133 (26.5%)	133 (26.5%)	
Gender, n (%)			0.687
Female	186 (37.1%)	181 (36.1%)	
Male	65 (12.9%)	70 (13.9%)	
Race, n (%)			0.341
Asian	26 (6.3%)	25 (6.1%)	
Black or African American	18 (4.4%)	9 (2.2%)	
White	174 (42.4%)	158 (38.5%)	
T stage, n (%)			**< 0.001**
T1	62 (12.4%)	81 (16.2%)	
T2	67 (13.4%)	97 (19.4%)	
T3	102 (20.4%)	68 (13.6%)	
T4	20 (4%)	3 (0.6%)	
N stage, n (%)			**< 0.001**
N0	96 (21.2%)	133 (29.4%)	
N1	134 (29.6%)	89 (19.7%)	
M stage, n (%)			0.183
M0	149 (51.2%)	133 (45.7%)	
M1	7 (2.4%)	2 (0.7%)	
Pathologic stage, n (%)			**< 0.001**
Stage I	132 (26.4%)	149 (29.8%)	
Stage II	12 (2.4%)	40 (8%)	
Stage III	70 (14%)	42 (8.4%)	
Stage IV	36 (7.2%)	19 (3.8%)	
Histological type, n (%)			**< 0.001**
Classical	198 (39.4%)	158 (31.5%)	
Follicular	20 (4%)	81 (16.1%)	
Other	2 (0.4%)	7 (1.4%)	
Tall Cell	31 (6.2%)	5 (1%)	
Residual tumor, n (%)			**0.014**
R0	185 (42%)	199 (45.2%)	
R1	35 (8%)	17 (3.9%)	
R2	3 (0.7%)	1 (0.2%)	
Extrathyroidal extension, n (%)			**< 0.001**
No	138 (28.5%)	193 (39.9%)	
Yes	105 (21.7%)	48 (9.9%)	
Primary neoplasm focus type, n (%)			**0.015**
Multifocal	100 (20.3%)	126 (25.6%)	
Unifocal	148 (30.1%)	118 (24%)	
Neoplasm location, n (%)			0.843
Bilateral	40 (8.1%)	46 (9.3%)	
Isthmus	12 (2.4%)	10 (2%)	
Left lobe	85 (17.1%)	90 (18.1%)	
Right lobe	109 (22%)	104 (21%)	
Thyroid gland disorder history, n (%)			**0.038**
Lymphocytic Thyroiditis	37 (8.3%)	34 (7.7%)	
Nodular Hyperplasia	23 (5.2%)	45 (10.1%)	
Normal	149 (33.6%)	131 (29.5%)	
Other, specify	12 (2.7%)	13 (2.9%)	
Living status, n (%)			0.799
Alive	242 (48.2%)	244 (48.6%)	
Dead	9 (1.8%)	7 (1.4%)	
Age, median (IQR)	46 (35, 58)	46 (35, 57)	0.680

### Correlation between *BTNL9* expression and immune cell infiltration

**Fig. 5:**
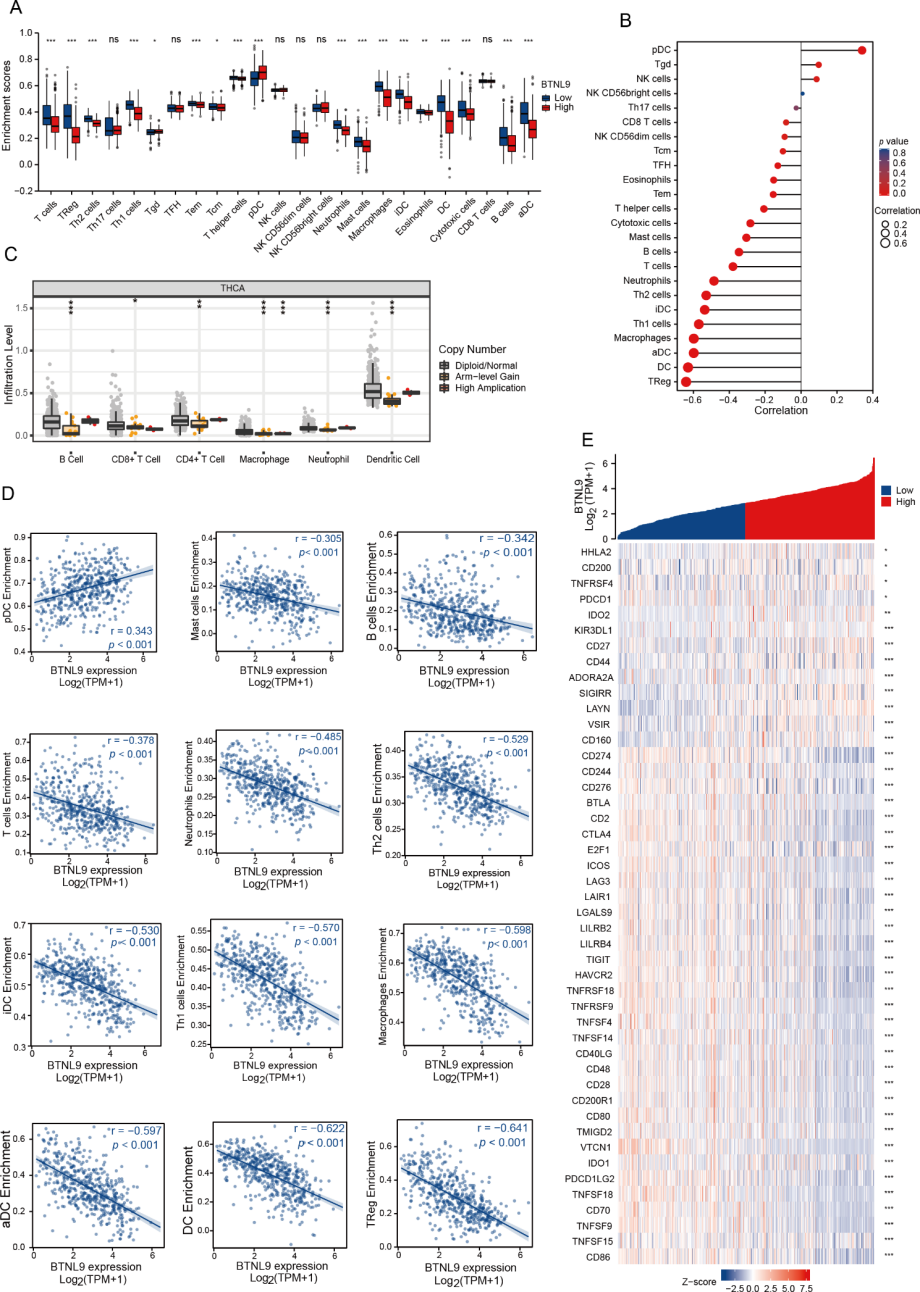
Association between *BTNL9* expression and immune infiltrations in THCA. **(A)** Box plot illustrating the enrichment of 24 subtypes of immune cells in high and low *BTNL9* expression groups. **(B)** Lollipop chart showing the correlation between *BTNL9* expression and different immune cells. **(C)***BTNL9* copy number variations were related to the infiltration levels of B cells, CD8 + T cells, CD4 + T cells, macrophages, neutrophils and dendritic cells in THCA. **(D)** Correlations between *BTNL9* expression and infiltration levels of different immune cells. **(E)** Heatmap indicating correlations between *BTNL9* expression and immune checkpoint genes. THCA, thyroid carcinoma; Tcm cell, central memory T cell; Treg cell, regulatory T cell; Th1 cell, T helper 1 cell; Th2 cell, T helper 2 cell; Tem cell, effector memory T cell; TFH cell, follicular helper T cell; NK cell, natural killer cell; DC, dendritic cells; pDC, plasmacytoid dendritic cells; iDC, interdigitating cell; aDC, activated dendritic cells. *p < 0.05, **p < 0.01, ***p < 0.001

The tumor microenvironment components played a vital role in THCA, exerting both antitumor and tumor-promoting functions [26–31]. The enrichment levels of 24 types of immune cells were assessed between groups of high and low *BTNL9* expression, and it revealed that T cells, Treg cells (regulatory T cell), Th1 cells (T helper 1 cell), Th2 cells (T helper 2 cell), T helper cells, Tem cells (effector memory T cell), Tcm cells (central memory T cell), neutrophils, mast cells, macrophages, eosinophils, DC (dendritic cells), iDC (interdigitating cell), aDC (activated dendritic cells), cytotoxic cells and B cells were significantly enriched in low *BTNL9* expression group (Fig. 5A).

Besides, it revealed that *BTNL9* expression was positively correlated with pDC (plasmacytoid dendritic cells, r = 0.343, *p* < 0.001), Tgd cells (r = 0.098, *p* = 0.028) and NK cells (natural killer cells, r = 0.095, *p* = 0.032). In contrast, negative correlations were found between *BTNL9* expression and enrichment levels of NK CD56dim cells (r =-0.092, *p* = 0.038), Tcm cells (r = -0.101, *p* = 0.024), TFH cells (follicular helper T cells, r = -0.129, *p* = 0.004), eosinophils (r = -0.153, *p* < 0.001), Tem cells (effector memory T cells, r = -0.155, *p* < 0.001), T helper cells (r = -0.21, *p* < 0.001), cytotoxic cells (r = -0.282, *p* < 0.001), mast cells (r = -0.305, *p* < 0.001), B cells (r = -0.342, *p* < 0.001), T cells (r = -0.378, *p* < 0.001), neutrophils (r = -0.485, *p* < 0.001), Th2 cells (r = -0.529, *p* < 0.001), iDC (r = -0.53, *p* < 0.001), Th1 cells (r = -0.57, *p* < 0.001), macrophages (r = -0.598, *p* < 0.001), aDC (r = -0.597, *p* < 0.001), DC (r = -0.622, *p* < 0.001), Treg cells (r = -0.641, *p* < 0.001) (Fig. 5B,D).

*BTNL9* expression showed strong relationships with a large proportion of chemokines/cytokines (**Additional file 1: Table ****S1**), which indicated that *BTNL9* expression might get involved in the immune-related biological processes in coordination with these genes. Copy number variations of *BTNL9* gene were found to have associations with the infiltration levels of B cells, CD8 + T cells, CD4 + T cells, macrophages, neutrophils and dendritic cells in THCA (Fig. 5C). The associations between *BTNL9* expression and common immune checkpoint genes were explored in THCA, and most of the immune checkpoint genes were significantly negatively correlated with *BTNL9* expression (Fig. 5E).

**Fig. 6:**
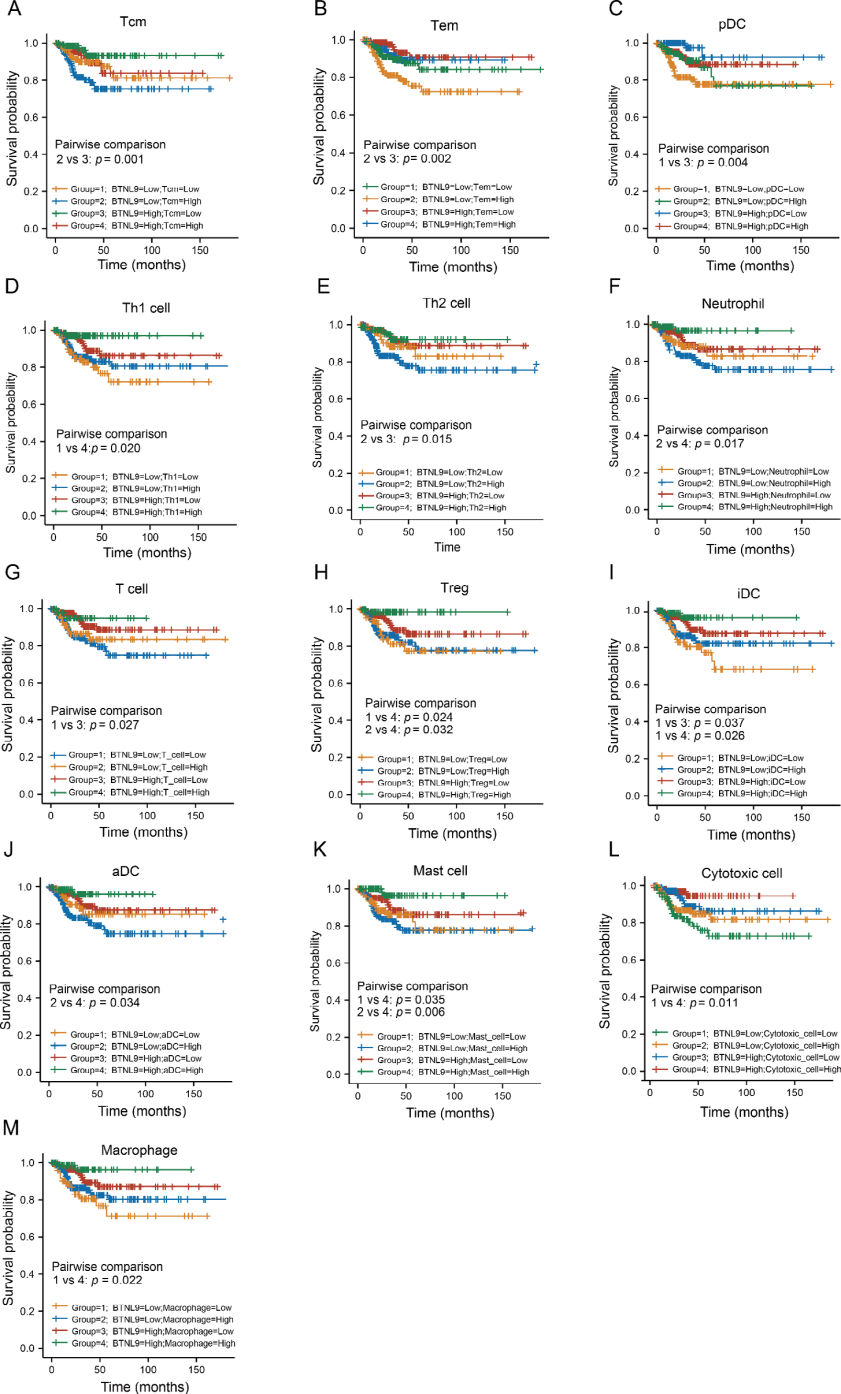
Kaplan-Meier survival curves of PFI stratified by immune cell scores and *BTNL9* expression in THCA. PFI curves analyzed with the combination of *BTNL9* expression level and **(A)** Tcm cell scores, **(B)** Tem cell scores, **(C)** pDC scores, **(D)** Th1 cell scores, **(E)** Th2 cell scores, **(F)** neutrophil cell scores, **(G)** T cell scores, **(H)** Treg cell scores, **(I)** iDC scores, **(J)** aDC scores, **(K)** mast cell scores, **(L)** cytotoxic cell scores and **(M)** macrophage cell scores. PFI, progress-free interval; THCA, thyroid carcinoma; Tcm cell, central memory T cell; Treg cell, regulatory T cell; Th1 cell, T helper 1 cell; Th2 cell, T helper 2 cell; Tem cell, effector memory T cell; pDC, plasmacytoid dendritic cells; iDC, interdigitating cell; aDC, activated dendritic cells

Furthermore, the data of *BTNL9* expression combined with immune cell infiltration from TCGA dataset was explored by Kaplan-Meier analysis. It demonstrated that in THCA, patients with high *BTNL9* expression and low infiltration scores of Tcm cells, Tem cells or pDC showed favorable outcomes of PFI (Fig. 6A-C), whereas those with high *BTNL9* expression combined with high scores of Th1 cells, Th2 cells, neutrophils, T cells, Treg cells, iDC, aDC, mast cells, cytotoxic cells or macrophages (Fig. 6D-M) presented better PFI outcomes in THCA patients.

### Functional enrichment analysis of DEGs associated with *BTNL9* in THCA

**Fig. 7:**
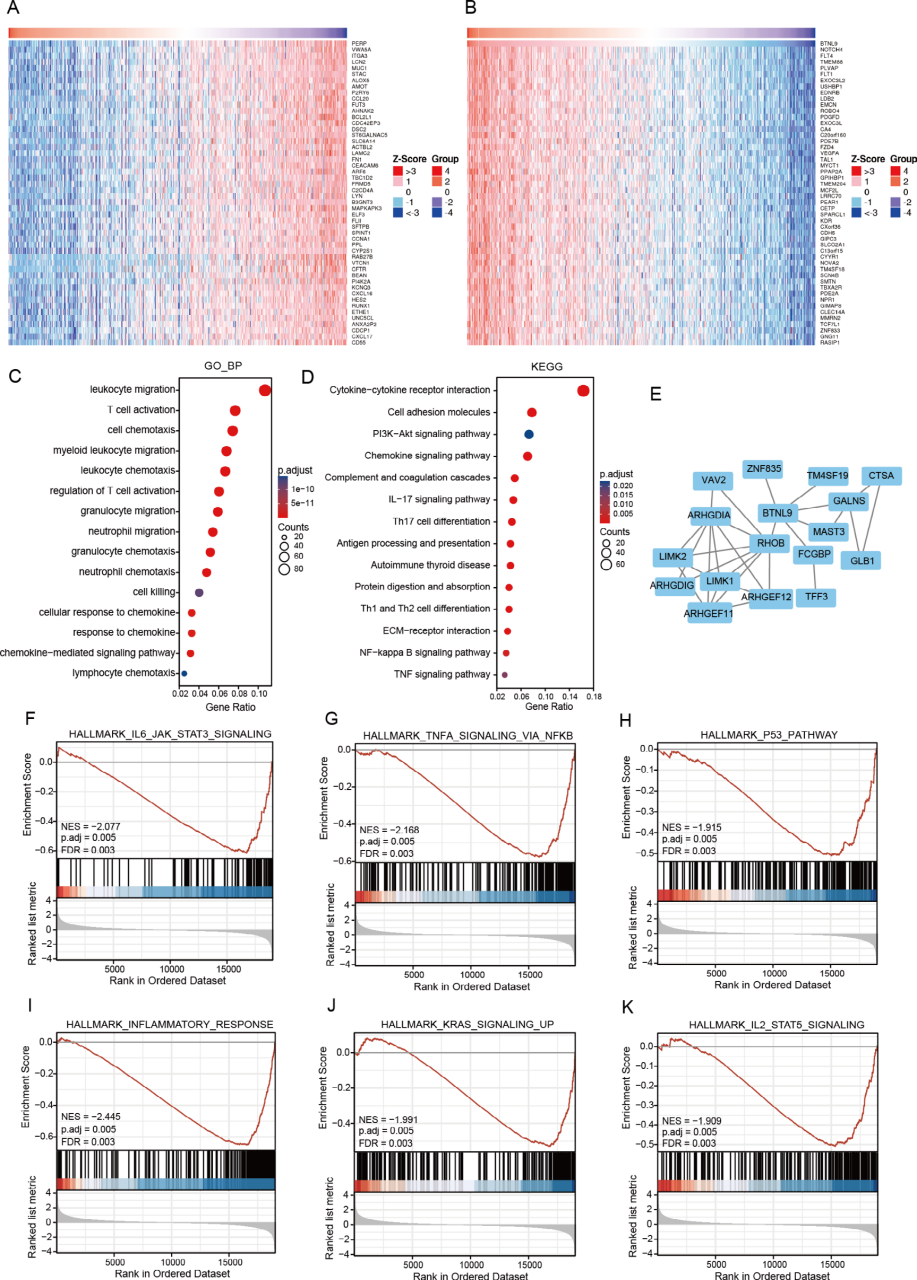
Functional enrichment analysis of differentially expressed genes (DEGs) associated with *BTNL9* gene in THCA. **(A)** Top 50 genes positively related with *BTNL9* in THCA. **(B)** Top 50 genes negatively related with *BTNL9* in THCA. **(C)** GO enrichment analysis of co-expressed genes. **(D)** KEGG pathway analysis [32] of co-expressed genes. **(E)** BTNL9 interaction network analysis using STRING tool. **(F-K)** GSEA plots showing DEGs enriched in immune-related pathways and cancer-related pathways. THCA, thyroid carcinoma; GO, gene ontology; KEGG, Kyoto Encyclopedia of Genes and Genomes; GSEA, gene set enrichment analysis

A total of 1595 DEGs associated with *BTNL9* were identified in THCA, including 733 up-regulated and 862 down-regulated genes (**Additional file 2: Table ****S2**). Besides, the co-expression network of *BTNL9* gene was explored by the Linkedomics platform with top 50 positively-related genes and negatively-related genes visualized in the forms of heatmaps (Fig. 7A, B).

Enrichment analyses of the DEGs were performed. GO annotations revealed that related genes were mainly involved in immune-related pathways such as leukocyte migration, T cell activation and cell chemotaxis (Fig. 7C). Similarly, KEGG enrichment analysis [32] revealed that these genes were mostly enriched in immune functions such as chemokine signaling pathway, complement and coagulation cascades, and IL-17 signaling pathway (Fig. 7D). The detailed pathway analysis was presented in **Additional file 2: Table ****S2**. GSEA analysis showed these genes were enriched in tumorigenesis and immune-related pathways, such as TNFα -signaling via NF-kappa B, P53 pathway, and inflammatory response (Fig. 7F-K). In addition, STRING online platform was used to investigate the protein–protein interaction (PPI) network, and 6 proteins were found to have direct interactions with BTNL9 (Fig. 7E).

### Prognostic analysis of clinical indicators in thyroid cancer

**Fig. 8:**
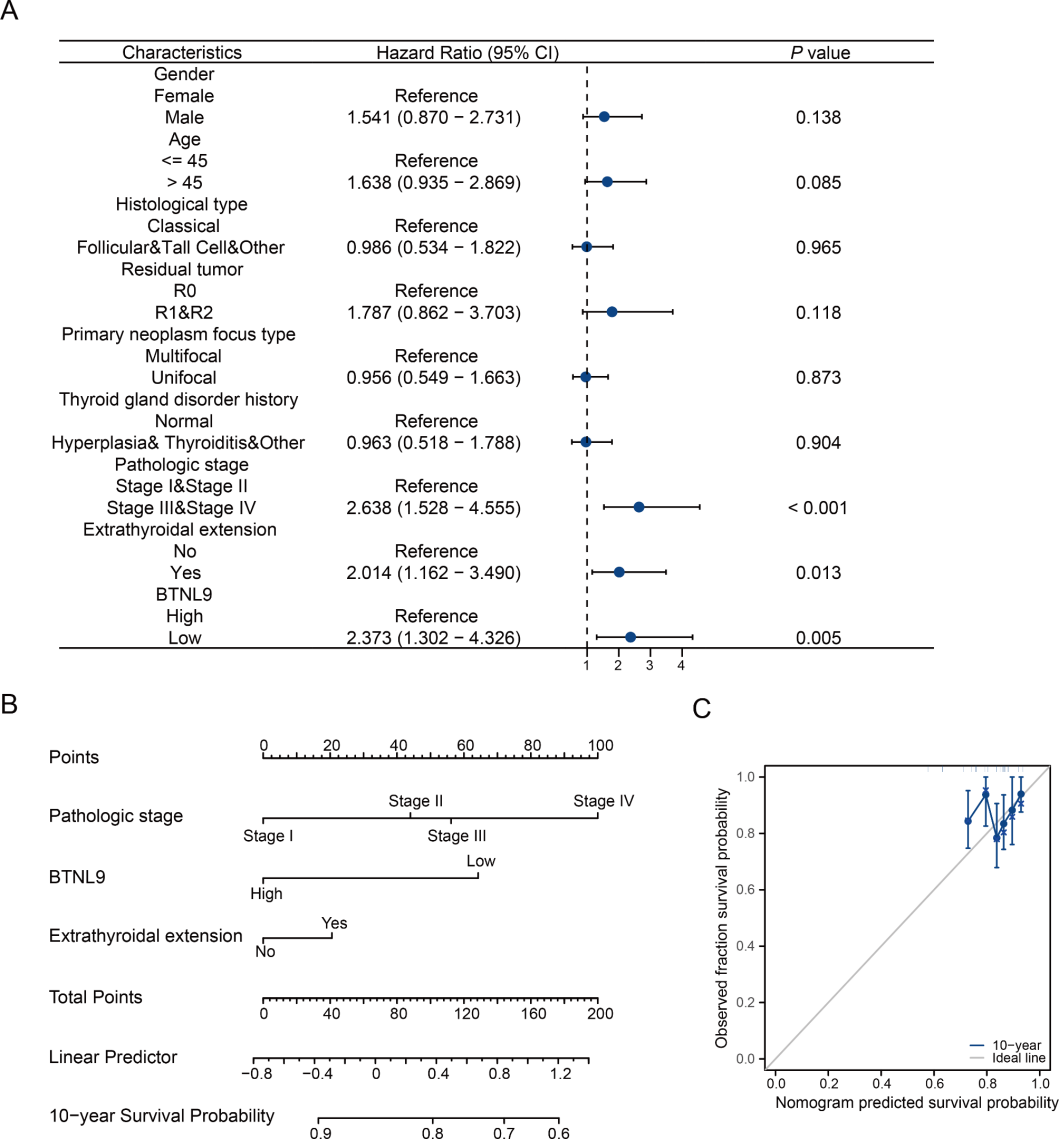
Prognostic prediction analysis of THCA. **(A)** Forest plot for univariate analysis of PFI in THCA based on TCGA datasets. **(B)** Nomogram plot predicting the probability of 10-year PFI in THCA patients. **(C)** Calibration plot for 10-year PFI prediction. THCA, thyroid carcinoma; PFI, progress-free interval

Cox regression analysis was performed to study the indicators of PFI in THCA. It showed that in univariate model, pathologic stage, tumor extrathyroidal extension and *BTNL9* expression were related to PFI (Fig. 8A) (Table 2). Low *BTNL9* expression is associated with higher hazard ratio of PFI in THCA. The factors with statistical significance in univariate analysis were then enrolled into multivariate Cox regression, and it turned out that *BTNL9* expression and pathologic stage were independent prognostic indicators (Table 2). The risk score list of the Cox regression analysis was provided in **Additional file 3: Table ****S3****.** Furthermore, nomogram model was constructed to predict 10-year PFI in THCA patients using the above clinical features, with the calibration curve evaluating the consistency between predicted probability and ideal model plotted (Fig. 8B-C).

**Table 2:** Cox regression analysis of prognostic factors for progression free interval in thyroid cancer

Characteristics	Univariate analysis			Multivariate analysis	
	Hazard ratio (95% CI)	*p* value		Hazard ratio (95% CI)	*p* value
Age (≤ 45 vs. >45)	1.618 (0.935–2.869)	0.085			
Gender (Female vs. Male)	1.541 (0.870–2.731)	0.138			
Histological type (Classical vs. Follicular &Tall Cell& Other)	0.986 (0.534–1.822)	0.965			
Residual tumor (R0 vs. R1&R2)	1.787 (0.862–3.703)	0.118			
Primary neoplasm focus type (Multifocal vs. Unifocal)	0.956 (0.549–1.663)	0.873			
Thyroid gland disorder history (Normal vs. Nodular Hyperplasia & Lymphocytic Thyroiditis & Other)	0.963 (0.518–1.788)	0.904			
BTNL9 (high vs. low)	2.373 (1.302–4.326)	0.005		1.931 (1.042–3.580)	0.037
Pathologic stage (StageI & StageII vs. StageIII & StageIV)	2.638 (1.528–4.555)	< 0.001		2.015 (1.082–3.751)	0.027
Extrathyroidal extension (No vs. Yes)	2.014 (1.162–3.490)	0.013		1.279 (0.687–2.381)	0.437

## Discussion

Cancer biomarkers, which included genetic alterations, epigenetic changes, proteins and pathological features, had been used in facilitating accurate diagnosis and prognostic evaluations in THCA, providing information for precise clinical management. It is recommended by American Thyroid Association guidelines that molecular testing is useful in making appropriate treatment regimen and reducing unnecessary surgical operation [4, 33]. With the development of high-throughput screening, more novel biomarkers were identified. In this study, by integrating H3K27ac ChIP-seq and RNA-seq data, *BTNL9* was found to have stronger H3K27ac signals with higher expression in BTN samples than PTC samples. The expression differences were further validated in PTC and corresponding adjacent normal tissues. Besides, high *BTNL9* expression in THCA was found to be related with favorable PFI in THCA based on TCGA datasets, and comprehensive bioinformatics analysis was conducted, suggesting the potential value of *BTNL9* in differential diagnosis of thyroid nodules and clinical prognosis.

Epigenetic alterations greatly impact cancer development. H3K27ac is an established mark of transcriptional activation. Our data presented epigenetic regulations of *BTNL9* gene with differential expression spectrums in PTC and BTN samples. Besides from THCA, decreased expression levels of *BTNL9* gene were also presented in various cancers according to TCGA datasets, which is consistent with previous studies [12–16]. Kaplan-Meier analysis suggested the prognostic value of *BTNL9* in THCA. Analyses of *BTNL9* expression in different clinical and pathologic stages showed decreased expression in more advanced stages, indicating *BTNL9* might participate in tumor progression. As for clinical parameters, lower *BTNL9* expression was shown in tall cell histological type, unifocal neoplasms, R1 and R2 residual tumors, and lymphocytic thyroiditis compared with other subtypes. The results indicated the potential involvement of *BTNL9* in tumorigenesis and progression as the above clinical factors were regarded as established risk factors for THCA [34, 35].

The tumor microenvironment components played a vital role in tumor initiation, progression and metastasis [36]. Numerous immune cells infiltrated the THCA microenvironment, exerting both antitumor and promoting functions, and in some way influence patients’ outcomes [26–31]. A number of subsets of immune cells were found to have effects on THCA and be correlated with disease outcomes. It was reported that immature DC subsets functioned as immune suppressor in tumor progression while matured DC showed anti-tumor effects [37]. Treg cells shut down antitumor immune response and promote tumor growth, and increased Treg cells in metastatic lymph nodes were associated with aggressive THCA [38], while in PTC a higher Treg cell infiltration was related to disease progression, extrathyroidal extension and recurrence [38–40]. Mast cell infiltration was associated with extrathyroidal extension in PTC [28]. Tumor-associated neutrophil density was correlated with tumor size in THCA samples, indicating the potential of promoting cancer growth [41]. High density of tumor-associated macrophages was related to more malignant biological behaviors, including lymph node metastasis in PTC [42], decreased survival in advanced THCA [27], and distant lung metastasis [31]. BTNL9 was shown to have negative correlations with these immune cells, indicating it might get involved in immunity microenvironment modulation. Immune-related cytokines and chemokines were investigated in THCA, and some of them exerted tumor-promotion effect. CCL2 was reported to promote lymph node metastasis and recurrence in PTC [43], and IL-10 was related to tumor size and invasion in THCA [44]. FOXP3 was found to have associations with DTC aggressiveness and tumor diameter [45]. The correlations between BTNL9 and these cytokines/chemokines indicated its involvement in tumor immunity process. The immune checkpoint PD-1/PD-L1 axis (CTLA-4, TIGIT, TIM3) activation played an important part in thyroid carcinogenesis [46]. PD-1 expression was related to DTC aggressiveness and unfavorable survival outcomes in THCA patients [47, 48]. Previous study showed that PD-1 inhibitor could be used in the treatment of ATC [49], and several ongoing clinical trials are evaluating the effects of combining PD-1 inhibitor with other therapies in advanced THCA treatment. *BTNL9* expression was negatively related with many immune checkpoint genes including PD-1 axis, suggesting the possibility of BTNL9 as a potential therapeutic target in THCA.

Furthermore, the functional enrichment analysis showed BTNL9 was involved in the immune-related and tumor-related regulating signaling pathways, indicating that low expression of BTNL9 might promote cancer development in THCA. Cox regression analysis suggested that *BTNL9* expression was an independent prognostic indicator in THCA, and a nomogram model incorporating *BTNL9* expression was established to predict 10-year PFI, which could provide a clinical reference for identifying high-risk patients. However, some limitations should be pointed out. First, the analyses were mainly performed by bioinformatics method based on public database, and further validation experiments need to be conducted. Second, the mechanisms by which BTNL9 take part in immune regulation remained unclear and warrant further in vivo and in vitro studies.

## Conclusion

In summary, the epigenetic H3K27ac modifications and expression levels of *BTNL9* gene were decreased in THCA. *BTNL9* expression was an independent prognostic indicator of PFI, and low expression was related to unfavorable prognosis in THCA patients. The *BTNL9* expression was closely associated with various immune cell infiltrations and immune checkpoints. The DEGs associated with *BTNL9* were mainly involved in immune-related and cancer-related pathways. The analyses raised the possibility of BTNL9 being a novel prognostic biomarker and a potential therapeutic target in THCA.

### Electronic supplementary material

Below is the link to the electronic supplementary material.

Supplementary Material 1

Supplementary Material 2

Supplementary Material 3

## Data Availability

The datasets presented in this study can be found in online repositories. The names of the repositories and accession number can be found in the article. TCGA : https://portal.gdc.cancer.gov/, GTEx: https://gtexportal.org/, GEPIA2: http://gepia.cancer-pku.cn/, Kaplan-Meier plotter: http://kmplot.com, TIMER2.0: http://timer.cistrome.org/, Linked Omics: http://www.linkes.org/login.php, STRING: https://cn.string-db.org/. H3K27ac ChIP-seq and RNA-seq data can be found in Genome Sequence Archive (GSA): https://ngdc.cncb.ac.cn/gsa/ (Accession number HRA000779).
